# Synthesis and evaluation of novel spiro derivatives for pyrrolopyrimidines as anti-hyperglycemia promising compounds

**DOI:** 10.1080/14756366.2018.1461854

**Published:** 2018-04-30

**Authors:** Samar Said Fatahala, Shahenda Mahgub, Heba Taha, Rania Helmy Abd-El Hameed

**Affiliations:** a Pharmaceutical Organic Chemistry Department, Helwan University, Helwan, Cairo, Egypt;; b Biochemistry Department, Faculty of Pharmacy, Helwan University, Helwan, Cairo, Egypt

**Keywords:** Spiropyrimidines, pyrrolopyrimidines, anti-hyperglycemic assay

## Abstract

Pyrrolopyrimidin-4-ylidene-malononitriles **IIa**–**d** were prepared as important intermediates for preparation of a new series of spiro-pyrrolopyrimidines. These intermediates undergo cyclisation *via* reaction with acetylacetone, guanidine hydrochloride or hydrazine hydrate. Elemental and spectroscopic evidences for the structures of these compounds are presented. The final compounds have been monitored for *in vivo* anti-hyperglycemic activity, compared with Amaryl as standard drug. Among 12 tested compounds, both spiro (pyrano **IIIb** and pyrazlo **Va)** derivatives exhibit promising anti-hyperglycemic activity.

## Introduction

Diabetes mellitus (DM) is a severe metabolic complaint that has a significant influence on the health and feature of patients’ life^1^. In 2013, 382 million adults were diagnosed with diabetes worldwide. This number is expected to grow to 592 million in 2035, of which 90% will have type 2 diabetes (non-insulin-dependent diabetes mellitus; T2D)^2^. Patients with T2D are 2–4 times more likely to have fatal or non-fatal coronary events or a stroke. Almost 70–80% of patients die from one of these two conditions. The International Diabetes Federation (IDF) listed Egypt among the world top 10 countries in the number of patients with diabetes^3–5^. In Egypt, the predominance of diabetes is around 15.56% among adults (age: 20 and 79 years), with an annual death of 86,478 related to diabetes^2^.

**Figure 1.**
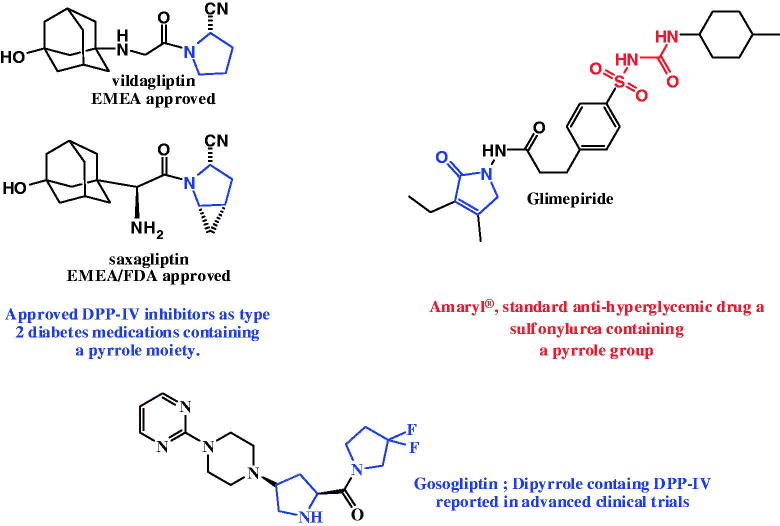
Pyrroles and pyrrolopyrimidines as anti-diabetic agents.

Treatment of diabetic patients has been concentrated on dietary controlling and well-known anti-hyperglycemic like sulfonylureas, metformin and acarbose. Glimepiride (Amaryl^®^, Sanofi-Aventis, Gentilly, France), a sulfonylurea containing a pyrrole group, acting as anti-hyperglycemic drug^6^. It indicated to treat type 2 diabetes through increase insulin production by the pancreas (Figure 1). Recently, urgent requisite to develop novel anti-hyperglycemic agents was observed^7^^,^^8^.

Numerous adverse effects present anti-hyperglycemic were indicated such as hepatotoxicity, weight gain and hypoglycemia^9^. Administration of dipeptidyl peptidase IV (DPP-IV)^10–13^ inhibitors to diabetic patients results in higher concentrations of endogenous glucagon-like peptide (GLP-1) lead to decrease in plasma glucose. Long-term treatment with a DPP-IV inhibitor reduced HbA1c (glycosylated haemoglobin), offered prospective improvement in insulin producing function of the pancreas.

DPP-IV inhibitors^14^ were validated to be active and safe compounds that control blood glucose. Vildagliptin, saxagliptin, DPP-IV inhibitors, (having pyrrole and fused pyrrole ring^15^^,^^16^, are on the market in many countries. Gosogliptin, di-pyrrole containing DPP-IV inhibitors, has been reported in advanced clinical trials. A highly potent DPP-IV inhibitor with pyrrolopyrimidine was also reported^17^^,^^18^ (Figure 1).

**Figure 2.**
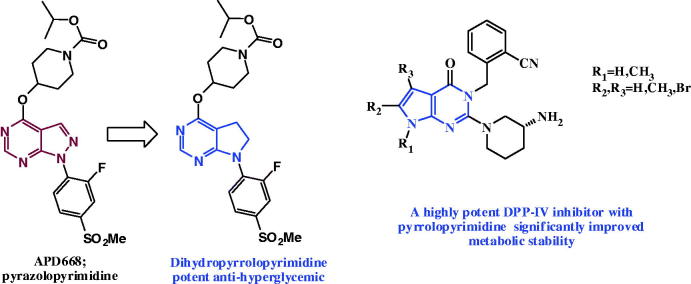
Pyrrolopyrimidines as anti-diabetic agents.

In 2004, pyrazolopyrimidine **APD668** was discovered by Arena pharmaceutics, was found to exhibition high *in vivo* activity compared to a known DPP-IV inhibitor. **APD668** was found to be more potent on delaying the onset of hyperglycemia (Figure 2). Researchers at GlaxoSmithKline replacement of pyrazolopyrimidine ring system in **APD668** with a dihydropyrrolopyrimidine scaffold, which were described as having therapeutic value for diabetes and associated conditions, obesity, glucose intolerance, insulin resistance and atherosclerosis^19^ (Figure 2).

**Figure 3.**
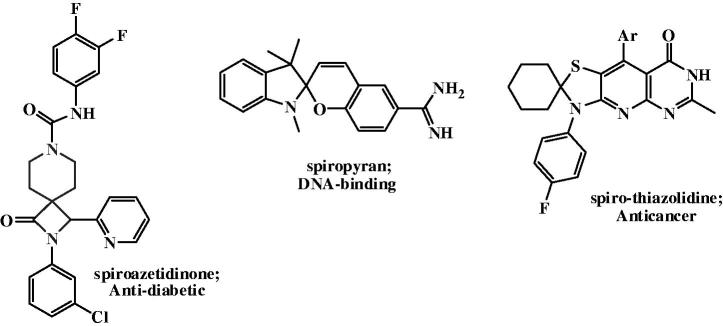
Spiro compounds as biological active scaffolds.

Spiro-based heterocyclic systems^20^, containing one carbon atom common to two rings, were found to be very motivating^21^. The asymmetric nature of these compounds, due to the spiro carbon, found to be one of the important criteria of the biological activities^22–26^. Several patents described spiroazetidine and spiroazetidinone derivatives as GPR119 receptor agonists for the treatment of diabetes^19^ (Figure 3).

**Scheme 1.**
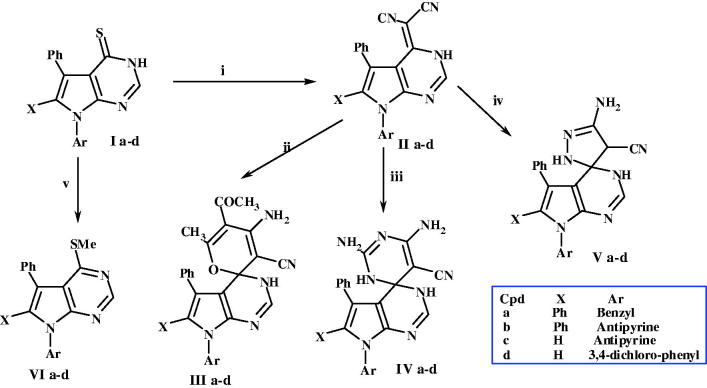
Synthetic pathway for preparation of II-V [reagents; i = NC-CH_2_-CN, ii = (CH_3_CO)_2_CH_2_, iii = (NH_2_)_2_C = NH, iv = NH_2_NH_2_, v = MeI].

Encouraged by the prominence of spiro containing compounds, and in maintenance of our research efforts^27–31^, in this research, we are going to spot an aspect on the chemistry of some newly synthesised spiro-pyrrolopyrimidine derivatives and estimate them for the anti-diabetic activities. The synthetic pathways approved for the synthesis of these compounds are revealed in Scheme 1.

## Materials and methods

### Synthesis of lead compounds

All commercial chemicals used as starting materials and reagents in this study were purchased from Merck (Darmstadt, Germany) and were of reagent grade. All melting points were uncorrected and measured using Electro-thermal IA 9100 apparatus (Shimadzu, Japan); IR spectra were recorded as potassium bromide pellets on a Perkin-Elmer 1650 spectrophotometer (Waltham, MA, USA), Faculty of Science, Cairo University, Cairo, Egypt. ^1^H NMR spectra were determined on a Varian Mercury (300 MHz) spectrometer (Varian, UK) and chemical shifts were expressed as ppm against TMS as internal reference (Faculty of Science, Cairo University, Cairo, Egypt). Mass spectra were recorded on 70 eV (EI Ms-QP 1000 EX, Shimadzu, Japan), Faculty of Science, Cairo University, Cairo, Egypt. Microanalyses were operated using Vario, Elementary apparatus (Shimadzu, Japan), Organic Microanalysis Unit, Faculty of Science, Cairo University, Cairo, Egypt. Column Chromatography was performed on (Merck) Silica gel 60 (particle size 0.06–0.20 mm). Compounds **Ia**–**d** were synthesised as reported^32–34^. The rest of compounds prepared in this paper were new and their structures were confirmed using spectral data.

#### *General procedure for the synthesis of compounds IIa*–*d*

Compounds **Ia**–**d** (0.01 mol) and malononitrile (0.66 g, 0.01 mol) were heated under reflux in dry ethanol (30 ml) for 8 h, cooled, poured onto ice-water to give precipitate which was filtered off, dried and recrystallised from methanol to give **IIa**–**d**.

##### 2-(7-benzyl-5,6-diphenyl-3H-pyrrolo[2,3-d]pyrimidin-4-ylidene)-malononitrile (IIa)

Yield: 73%; m.p.: 179–181 °C; IR (KBr) *υ* (cm^−1^): 3318 (N–H), 2219 (C≡N), 1607 (C=N); MS (EI) *m/z*: 425 (M^+^, 67%), ^1^H NMR (DMSO-d_6_, 300 MHz) *δ* (ppm): 5.4 (s, 2H, Ph–CH_2_), 6.8–8.0 (m, 15H, Ar–H ), 8.18 (s, 1H, C2–H), 8.9 (s, 1H, NH, D_2_O exchangeable); Anal. Calcd for C_28_H_19_N_5_ (425.16): C, 79.06; H, 4.47; N, 16.47%. Found: C, 79.38; H, 4.66; N, 16.07%.

##### 2-(7-(1,5-dimethyl-3-oxo-2-phenyl-2,3-dihydro-1H-pyrazol-4-yl)-5,6-diphenyl-7H-pyrrolo[2,3-d]pyrimidin-4-ylidene)-malononitrile (IIb)

Yield: 67%; m.p.: 186–188 °C; IR (KBr) *υ* (cm^−1^): 3287 (N–H), 2228 (C≡N), 1693 (C=O), 1608 (C=N); MS (EI) *m/z*: 521 (M^+^, 28%), ^1^H NMR (DMSO-d_6_, 300 MHz) *δ* (ppm): 2.25 (s, 3H, CH_3_), 3.5 (s, 3H, NCH_3_), 6.6–7.8 (m, 15H, Ar–H ), 8.12 (s, 1H, C2–H), 8.9. (s, 1H, NH, D_2_O exchangeable); Anal. Calcd for C_32_H_23_N_7_O (521.57): C, 73.70; H, 4.41; N, 18.81%. Found: C, 74.01; H, 4.35; N, 18.92%.

##### 2-(7-(1,5-dimethyl-3-oxo-2-phenyl-2,3-dihydro-1H-pyrazol-4-yl)-5-phenyl-7H-pyrrolo[2,3-d]pyrimidin-4-ylidene)-malononitrile (IIc)

Yield: 54%; m.p.: 173–175 °C; IR (KBr) *υ* (cm^−1^): 3295 (N–H), 2211 (C≡N), 1691 (C=O), 1588 (C=N); MS (EI) *m/z*: 445 (M^+^, 73.4%), ^1^H NMR (DMSO-d_6_, 300 MHz) *δ* (ppm): 2.2 (s, 3H, CH_3_), 3.42 (s, 3H, NCH_3_), 6.6–7.8 (m, 11H, Ar–H ), 8.5 (s, 1H, C2–H), 8.9 (s, 1H, NH, D_2_O exchangeable); Anal. Calcd for C_26_H_19_N_7_O (445.48): C, 70.11; H, 4.27; N, 22.02%. Found: C, 70.38; H, 4.61; N, 22.28%.

##### 2-(7-(3,4-dichlorophenyl)-5-phenyl-7H-pyrrolo[2,3-d]pyrimidin-4-ylidene)-malononitrile (IId)

Yield: 56%; m.p.: 191–193 °C; IR (KBr) *υ* (cm^−1^): 3347 (N–H), 2212 (C≡N), 1581 (C=N); MS (EI) *m/z*: 404 (M^+^, 13.5%), 406 (M^+^+2, 8.5%), 408 (M^+^+4, 2.7%) ^1^H NMR (DMSO-d_6_, 300 MHz) *δ* (ppm): 6.8–7.8 (m, 9H, Ar–H ), 8.09 (s, 1H, C2–H), 8.83 (s, 1H, NH, D_2_O exchangeable); Anal. Calcd for C_21_H_11_Cl_2_N_5_ (404.25): C, 62.38; H, 2.72; N, 17.33%. Found: C, 62.05; H, 2.69; N, 17.71%.

#### General procedure for the synthesis of compounds IIIa–d

A mixture of compounds **IIa**–**d** (0.02 mol), acetylacetone (2g, 0.02 mol) and pyridine (6–8 drops) was heated under reflux in dry ethanol (50 ml) for 8 h, concentrated, cooled and the separated compound was filtered off and recrystallised from methanol to give **IIIa**–**d**.

##### 5-acetyl-4-amino-7′-benzyl-6-methyl-5′,6′-diphenyl-spiro[3H-pyrrolo[2,3-d]pyrimidine-4′,2-pyran]-3-carbonitrile (IIIa)

Yield: 72%; m.p.: 187–189 °C; IR (KBr) *υ* (cm^−1^): 3212–3345 (N–H, NH_2_), 2199 (C≡N), 1667 (C=O), 1598 (C=N), 1260 (C–O); MS (EI) *m/z*: 525 (M^+^, 41%), ^1^H NMR (DMSO-d_6_, 300 MHz) *δ* (ppm): 2.23(s, 3H, C6–CH_3_), 2.27(s, 3H, COCH_3_), 5.8(s, 2H, Ph–CH_3_), 4.7 (brs, 2H, NH_2_, D_2_O exchangeable), 6.9–7.7 (m, 15H, Ar–H), 8.3 (s, 1H, C2*′*–H). 8.8 (s, 1H, NH, D_2_O exchangeable); Anal. Calcd for C_33_H_27_N_5_O_2_ (525.60): C, 75.43; H, 5.14; N, 13.33%. Found: C, 75.80; H, 5.02; N, 13.61%.

##### 5-acetyl-4-amino-7′-(1,5-dimethyl-3-oxo-2-phenyl-2,3-dihydro-1H-pyrazol-4-yl)-6-methyl-5′,6′-diphenyl-spiro[3H-pyrrolo[2,3-d]pyrimidine-4′,2-pyran]-3-carbonitrile (IIIb)

Yield: 55%; m.p.: 195–197 °C; IR (KBr) *υ* (cm^−1^): 3250–3387 (N–H, NH_2_), 2207 (C≡N), 1678, 1693 (C=O), 1612 (C=N), 1270 (C–O); MS (EI) *m/z*: 621 (M^+^, 26%), ^1^H NMR (DMSO-d_6_, 300 MHz) *δ* (ppm): 2.2 (s, 3H, C6–CH_3_), 2.3(s, 3H, COCH_3_), 2.37 (s, 3H, CH_3_), 3.52 (s, 3H, NCH_3_), 5.2 (brs, 2H, NH_2_, D_2_O exchangeable), 6.9–7.9 (m, 15H, Ar–H), 8.1(s, 1H, C2*′*–H). 8.9 (s, 1H, NH, D_2_O exchangeable); Anal. Calcd for C_37_H_31_N_7_O_3_ (621.12): C, 71.50; H, 4.99; N, 15.78%. Found: C, 71.39; H, 5.18; N, 15.66%.

##### 5-acetyl-4-amino-7′-(1,5-dimethyl-3-oxo-2-phenyl-2,3-dihydro-1H-pyrazol-4-yl)-6-methyl-5′-phenyl-spiro[3H-pyrrolo[2,3-d]pyrimidine-4′,2-pyran]-3-carbonitrile (IIIc)

Yield: 65%; m.p.: 188–190 °C; IR (KBr) *υ* (cm^−1^): 3145–3348 (N–H, NH_2_), 2213 (C≡N), 1661, 1688 (C=O), 1602 (C=N), 1305 (C–O); MS (EI) *m/z*: 545 (M^+^, 31.4%), ^1^H NMR (DMSO-d_6_, 300 MHz) *δ* (ppm): 2.2 (s, 3H, C6–CH_3_), 2.34 (s, 3H, COCH_3_), 2.39 (s, 3H, CH_3_), 3.48 (s, 3H, NCH_3_), 4.82 (brs, 2H, NH_2_, D_2_O exchangeable), 7.0–7.9 (m, 11H, Ar–H), 8.3(s, 1H, C2*′*–H). 8.9 (s, 1H, NH, D_2_O exchangeable); Anal. Calcd for C_31_H_27_N_7_O_3_ (545.32): C, 68.26; H, 4.95; N, 17.98%. Found: C, 68.03; H, 5.20; N, 18.22%.

##### 5-acetyl-4-amino-7′-(3,4-dichlorophenyl)-6-methyl-5′-phenyl-spiro[3H-pyrrolo[2,3-d]pyrimidine-4′,2-pyran]-3-carbonitrile (IIId)

Yield: 33%; m.p.: 203–205 °C; IR (KBr) *υ* (cm^−1^): 3225–3419 (N–H, NH_2_), 2222 (C≡N), 1709 (C=O), 1614 (C=N), 1312 (C–O); MS (EI) *m/z*: 503 (M^+^, 60%), 505 (M^+^+2, 20.3%), 507 (M^+^+4, 8.3%) ^1^H NMR (DMSO-d_6_, 300 MHz) *δ* (ppm): 2.3 (s, 3H, C6–CH_3_), 2.41(s, 3H, COCH_3_), 4.8 (brs, 2H, NH_2_, D_2_O exchangeable), 6.9–7.8 (m, 9H, Ar-H), 8.4 (s, 1H, C2*′*–H). 8.9 (s,1H, NH, D_2_O exchangeable); Anal. Calcd for C_26_H_19_Cl_2_N_5_O_2_ (503.3): C, 62.03; H, 3.78; N, 13.92%. Found: C, 62.34; H, 3.52; N, 14.12%.

#### *General procedure for the synthesis of compounds IVa*–*d*

A mixture of compounds **IIa**–**d** (0.02 mol), guanidine (1.18g, 0.02 mol) and pyridine (6–8 drops) was heated under reflux in dry ethanol (50 ml) for 8 h, concentrated, cooled, and the separated compound was filtered off and recrystallised from methanol to give **IVa**–**d**.

##### 2,4-diamino-7′-benzyl-5′,6′-diphenyl-spiro[1H-pyrimidine-6,4′-3H-pyrrolo[2,3-d]pyrimidine]-5-carbonitrile (IVa)

Yield: 68%; m.p.: 195–197 °C; IR (KBr) *υ* (cm^−1^): 3126–3419 (N–H, NH_2_), 2212 (C≡N), 1618 (C=N); MS (EI) *m/z*: 484 (M^+^, 61%), ^1^H NMR (DMSO-d_6_, 300 MHz) *δ* (ppm): 5.26 (s, 2H, Ph–CH_2_), 4.2–4.6 (brs, 4H, 2NH_2_, D_2_O exchangeable), 6.9–8.1 (m, 16H, Ar–H + NH), 8.33 (s, 1H, C2*′*–H), 8.9 (s, 1H, NH, D_2_O exchangeable); Anal. Calcd for C_29_H_24_N_8_ (484.4): C, 71.90; H, 4.96; N, 23.14%. Found: C, 71.75; H, 4.98; N, 23.42%.

##### 2,4-diamino-7′-(1,5-dimethyl-3-oxo-2-phenyl-2,3-dihydro-1H-pyrazol-4-yl)-5′,6′-diphenyl-spiro[1H-pyrimidine-6,4′-3H-pyrrolo[2,3-d]pyrimidine]-5-carbonitrile (IVb)

Yield: 53%; m.p.: 197–199 °C; IR (KBr) *υ* (cm^−1^): 3153–3320 (N–H, NH_2_), 2227 (C≡N), 1698 (C=O), 1622 (C=N); MS (EI) *m/z*: 580 (M^+^, 23.9%), ^1^H NMR (DMSO-d_6_, 300 MHz) *δ* (ppm): 2.3 (s, 3H, CH_3_), 3.41 (s, 3H, NCH_3_), 4.2–4.5 (brs, 4H, 2NH_2_, D_2_O exchangeable), 7.0–7.7 (m, 16H, Ar–H + NH), 8.09 (s, 1H, C2*′*–H), 8.94 (s, 1H, NH, D_2_O exchangeable); Anal. Calcd for C_33_H_28_N_10_O (580.04): C, 68.28; H, 4.83; N, 24.14%. Found: C, 68.39; H, 5.09; N, 24.45%.

##### 2,4-diamino-7′-(1,5-dimethyl-3-oxo-2-phenyl-2,3-dihydro-1H-pyrazol-4-yl)-5′-phenyl-spiro[1H-pyrimidine-6,4′-3H-pyrrolo[2,3-d]pyrimidine]-5-carbonitrile (IVc)

Yield: 43%; m.p.: 194–196 °C; IR (KBr) *υ* (cm^−1^): 3239–3485 (N–H, NH_2_), 2205 (C≡N), 1682 (C=O), 1598 (C=N), MS (EI) *m/z*: 504 (M^+^, 22%), ^1^H NMR (DMSO-d_6_, 300 MHz) *δ* (ppm): 2.2 (s, 3H, CH_3_), 3.5 (s, 3H, NCH_3_), 4.05–4.4 (brs, 4H, 2NH_2_, D_2_O exchangeable), 6.8–7.8 (m, 12H, Ar–H + NH ), 8. 2 (s, 1H, C2*′*–H), 8.8 (s, 1H, NH, D_2_O exchangeable); Anal. Calcd for C_27_H_24_N_10_O (504.25): C, 64.29; H, 4.76; N, 27.78%. Found: C, 63.93; H, 4.65; N, 27.40%.

##### 2,4-diamino-7′-(3,4-dichlorophenyl)-5′-phenyl-spiro[1H-pyrimidine-6,4′-3H-pyrrolo[2,3-d]pyrimidine]-5-carbonitrile (IVd)

Yield: 35%; m.p.: 212–214 °C; IR (KBr) *υ* (cm^−1^): 3209–3345 (N–H, NH_2_), 2218 (C≡N), 1626 (C=N), MS (EI) *m/z*: 462 (M^+^, 58%), 464 (M^+^+2, 18.3%), 466 (M^+^+4, 5.7%), ^1^H NMR (DMSO-d_6_, 300 MHz) *δ* (ppm): 4.1– 4.4 (brs, 4H, 2NH_2_, D_2_O exchangeable), 6.9–8.0 (m, 10H, Ar–H + NH), 8.23 (s, 1H, C2*′*–H), 9.1 (s, 1H, NH, D_2_O exchangeable); Anal. Calcd for C_22_H_16_N_8_Cl_2_ (462.34): C, 57.14; H, 3.46; N, 24.24%. Found: C, 57.08; H, 3.62; N, 24.53%.

#### *General procedure for the synthesis of compounds Va*–*d*

A mixture of compound **IIa**–**d** (0.02 mol), hydrazine hydrate (0.64g, 0.02 mol) and pyridine (6–8 drops) was heated under reflux in dry ethanol (50 ml) for 8 h, concentrated, cooled, and the separated compound was filtered off and recrystallised from methanol to give **Va**–**d**.

##### 3-amino-7′-benzyl-5′,6′-diphenyl-spiro[1,4-dihydropyrazole-5,4′-3H-pyrrolo[2,3-d]pyrimidine]-4-carbonitrile (Va)

Yield: 72%; m.p.: 192–194 °C; IR (KBr) *υ* (cm^−1^): 3197–3342 (N–H, NH_2_), 2226 (C≡N), 1633 (C=N); MS (EI) *m/z*: 457 (M^+^, 29%), ^1^H NMR (DMSO-d_6_, 300 MHz) *δ* (ppm): 4.1(s, 1H, C–4H), 4.9 (s, 2H, Ph–CH_2_), 5.82 (s, 2H, NH_2_,D_2_O exchangeable), 6.8–7.9 (m, 16H, Ar–H + NH), 8.32 (s, 1H, C–2*′*H), 8.7(s,1H, NH, D_2_O exchangeable); Anal. Calcd for C_28_H_23_N_7_ (457.34): C, 73.52; H, 5.03; N, 21.44%. Found: C, 73.58; H, 4.80; N, 21.71%.

##### 3-amino-7′-(1,5-dimethyl-3-oxo-2-phenyl-2,3-dihydro-1H-pyrazol-4-yl)-5′,6′-diphenyl-spiro[1,4-dihydropyrazole-5,4′-3H-pyrrolo[2,3-d]pyrimidine]-4-carbonitrile (Vb)

Yield: 65%; m.p.: 201–203 °C; IR (KBr) *υ* (cm^−1^): 3139–3442 (N–H, NH_2_), 2216 (C≡N), 1688 (C=O), 1622 (C=N); MS (EI) *m/z*: 553 (M^+^, 91%), ^1^H NMR (DMSO-d_6_, 300 MHz) *δ* (ppm): 2.27 (s, 3H, CH_3_), 3.4 (s, 3H, NCH_3_), 4.3 (s, 1H, C–4H), 5.1 (s, 2H, NH_2_,D_2_O exchangeable), 7.0–8.0 (m, 16H, Ar–H + NH), 8.32 (s, 1H, C–2*′*H), 9.0 (s,1H, NH, D_2_O exchangeable); Anal. Calcd for C_32_H_27_N_9_O (553.39): C, 69.44; H, 4.88; N, 22.78%. Found: C, 69.80; H, 4.62; N, 22.45%.

##### 3-amino-7′-(1,5-dimethyl-3-oxo-2-phenyl-2,3-dihydro-1H-pyrazol-4-yl)-5′-phenyl-spiro[1,4-dihydropyrazole-5,4′-3H-pyrrolo[2,3-d]pyrimidine]-4-carbonitrile (Vc)

Yield: 53%; m.p.: 182–184 °C; IR (KBr) *υ* (cm^−1^): 3231–3448 (N–H, NH_2_), 2207 (C≡N), 1682 (C=O), 1633 (C=N); MS (EI) *m/z*: 477 (M^+^, 19.4%), ^1^H NMR (DMSO-d_6_, 300 MHz) *δ* (ppm): 2.31 (s, 3H, CH_3_), 3.38 (s, 3H, NCH_3_), 3.42 (s, 1H, C–4H), 4.4 (s, 2H, NH_2_,D_2_O exchangeable), 6.8–7.9 (m, 12H, Ar–H + NH), 8.35 (s, 1H, C–2*′*H), 8.9 (s,1H, NH, D_2_O exchangeable); Anal. Calcd for C_26_H_23_N_9_O (477.52): C, 65.41; H, 4.82; N, 26.42%. Found: C, 65.62; H, 4.71; N, 26.79%.

##### 3-amino-7′-(3,4-dichlorophenyl)-5′-phenyl-spiro[1,4-dihydropyrazole-5,4′-3H-pyrrolo[2,3-d]pyrimidine]-4-carbonitrile (Vd)

Yield: 39%; m.p.: 206–208 °C; IR (KBr) *υ* (cm^−1^): 3196–3335 (N–H, NH_2_), 2230 (C≡N), 1590 (C=N), MS (EI) *m/z*: 435 (M^+^, 32.7%), 437 (M^+^+2, 11.3%), 439 (M^+^+4, 3.9%), ^1^H NMR (DMSO-d_6_, 300 MHz) *δ* (ppm): 3.48 (s, 1H, C–4H), 4.2 (s, 2H, NH_2_, D_2_O exchangeable), 6.9–8.0 (m, 10H, Ar–H + NH), 8.48 (s, 1H, C2*′*–H), 8.91 (s, 1H, NH, D_2_O exchangeable); Anal. Calcd for C_21_H_15_N_7_Cl_2_ (435.28): C, 57.93; H, 3.45; N, 22.53%. Found: C, 57.78; H, 3.65; N, 22.16%.

#### *General procedure for the synthesis of compounds VIa*–*d*

A mixture of compounds **Ia**–**d** (0.02 mol) and methyl iodide (0.02 mol) was stirred in 10% NaOH solution at room temperature for 8 h, poured onto acidified ice-water to give a precipitate which was filtered off, dried and crystallised from methanol to afford compounds **VIa**–**d**.

##### 7-benzyl-4-methylsulfanyl-5,6-diphenyl-pyrrolo[2,3-d]pyrimidine (VIa)

Yield: 73%; m.p.: 187–189 °C; IR (KBr) *υ* (cm^−1^): 1583 (C=N); MS (EI) *m/z*: 407 (M^+^, 52.3%), ^1^H NMR (DMSO-d_6_, 300 MHz) *δ* (ppm): 3.12 (s, 3H, S–CH_3_), 4.97 (s, 2H, Ph–CH_2_), 6.9–7.8 (m, 15H, Ar–H), 8.3 (s, 1H, C–2H); Anal. Calcd for C_26_H_21_N_3_S (407.32): C, 76.66; H, 5.16; N, 10.32%. Found: C, 76.51; H, 4.93; N, 10.70%.

##### 7-(1,5-dimethyl-3-oxo-2-phenyl-2,3-dihydro-1H-pyrazol-4-yl)-4-methylsulfanyl-5,6-diphenyl-pyrrolo[2,3-d]pyrimidine (VIb)

Yield: 51%; m.p.: 183–185 °C; IR (KBr) *υ* (cm^−1^): 1708 (C=O), 1618 (C=N); MS (EI) *m/z*: 503 (M^+^, 73%), ^1^H NMR (DMSO-d_6_, 300 MHz) *δ* (ppm): 2.31 (s, 3H, CH_3_), 3.27 (s, 3H, S–CH_3_), 3.37 (s, 3H, N–CH_3_), 7.0–8.1 (m, 15H, Ar–H), 8.4 (s, 1H, C–2H); Anal. Calcd for C_30_H_25_N_5_OS (503.32): C, 71.57; H, 4.97; N, 13.92%. Found: C, 71.82; H, 4.66; N, 13.77%.

##### 7-(1,5-dimethyl-3-oxo-2-phenyl-2,3-dihydro-1H-pyrazol-4-yl)-4-methylsulfanyl-5-phenyl-pyrrolo[2,3-d]pyrimidine (VIc)

Yield: 49%; m.p.: 167–169 °C; IR (KBr) *υ* (cm^−1^): 1685 (C=O), 1614 (C=N); MS (EI) *m/z*: 427 (M^+^, 20.7%), ^1^H NMR (DMSO-d_6_, 300 MHz) *δ* (ppm): 2.27 (s, 3H, CH_3_), 3.2 (s, 3H, S–CH_3_), 3.4 (s, 3H, N–CH_3_), 7.0–7.9 (m, 11H, Ar–H), 8.2 (s, 1H, C–2H); Anal. Calcd for C_24_H_21_N_5_OS (427.3): C, 67.45; H, 4.92; N, 16.39%. Found: C, 67.69; H, 4.67; N, 16.55%.

##### 7-(3,4-dichlorophenyl)-4-methylsulfanyl-5,6-diphenyl-pyrrolo[2,3-d]pyrimidine (VId)

Yield: 41%; m.p.: 183–185 °C; IR (KBr) *υ* (cm^−1^): 1596 (C=N), MS (EI) *m/z*: 385 (M^+^, 22%), 387 (M^+^+2, 7.3%), 389 (M^+^+4, 2.6%), ^1^H NMR (DMSO-d_6_, 300 MHz) *δ* (ppm): 3.3 (s, 3H, S–CH_3_), 6.8–7.8 (m, 9H, Ar–H), 8.3 (s, 1H, C–2H); Anal. Calcd for C_19_H_13_N_3_SCl_2_ (385.32): C, 59.22; H, 3.38; N, 10.91%. Found: C, 59.61; H, 3.56; N, 11.13%.

## Biological screening

### Animals

The complete progress of the experiment was conducted using male Wistar albino rats (200–250 g), delivered by the Institutional Breeding House, Egypt, reared and maintained in the animal house of the institution. The animals had free access to food and water *ad libitum* and maintained in a controlled environment under standard conditions of temperature and humidity with an alternating 12 h light and dark cycle for about a week for acclimatisation. The protocol of the study was approved by the Animal Ethics Committee of the Faculty of Pharmacy, Helwan University on November 2016. The study was conducted in accordance with the EC, directive 86/609/EEC for animal experiments.

### Dose determination

Glimepiride (Amaryl) was used as a standard anti-diabetic (4 mg/kg) in 1% of gum acacia and administered orally^35^. Equivalent doses of all derivatives were calculated according to their molecular weight (M.wt).

#### Assessment of improvement on oral glucose tolerance and blood glucose lowering activity: sucrose loaded normal rats (SLM)

Male albino Wistar rats (200–250 g) were chosen and kept back on an overnight fasting. Next morning, the blood glucose level (0 min) of each animal was stated by glucometer using glucostrips. The animals presenting their fasting blood glucose levels in the range of 60–80 mg/dL were selected and separated into one control group and 13 experimental groups with six animals in each. Each rat of experimental groups was given suspension of the test compounds made in 1% of gum acacia at a dosage of (4 mg/kg) for the standard drug Glimepiride and Equivalent doses of all derivatives.

The animals of the control group received vehicle (1.0% of gum acacia) only. Exactly 30 min post-administration of the test samples/vehicle, an oral sucrose load of 10 g/kg body weight (bw) was given to each animal and the blood glucose level of each animal were measured at 30, 60, 90 and 120 min^36^. The percentages (%) decreased in blood glucose level were calculated conferring to the AUC method.

#### Streptozotocin-induced diabetic rats

Male albino Wister rats (200–250 g) were designated for this study. Diabetes was prompted in the rats by intraperitoneally (i.p.) injecting freshly prepared solution of Streptozotocin (STZ) (Sigma-Aldrich, Co., MO; catalogue number: 1001062761) in ice cold 0.1 M citrate buffer (pH 4.5)^37^ at a dosage of 50 mg/kg bw^38^. The blood glucose of each animal was tested after 48 h and animals displaying fasting blood glucose level ≥200 mg/dl were elected^39^. These diabetic rats were unsystematically scattered into groups consisting of six animals in each.

#### Experimental design

Five groups (eight rats each) were used to investigate the anti-hyperglycemic effect of the derivatives which showed promising anti-hyperglycemic effect in SLM (compounds **IIIa**, **Va** and **IIIb**). *Group 1:* diabetic control and *Group* 2: diabetic and Glimepiride (Amaryl) (4 mg/kg) served as a reference anti-diabetic drug. *Groups* 3–5 were given the various pyrrole derivatives (compounds **IIIa**, **Va** and **IIIb**). The treated groups administered the standard drug (Amaryl) and different derivatives orally. For each group, blood samples were collected by tail nipping and blood glucose level was estimated at 0, 1, 2, 4 and 6 h after oral administration of the tested compounds using glucometer (Gluco Dr Super Sensor, All Medicus Co., Ltd., Anyang, Gyeonggi, Korea).

#### Statistical analysis

Data were represented as mean area under curve (AUC) ± SD. Significant differences between groups was tested using GraphPad InStat (Graph software Inc., V 3.05, Ralph Stahlman, Purdue University, Lafayette, IN). Appropriate graphs were plotted using Microsoft Excel 2016. *p* Value less than .05 was considered statistically significant.

## Discussion

### Chemistry

**Figure 4.**
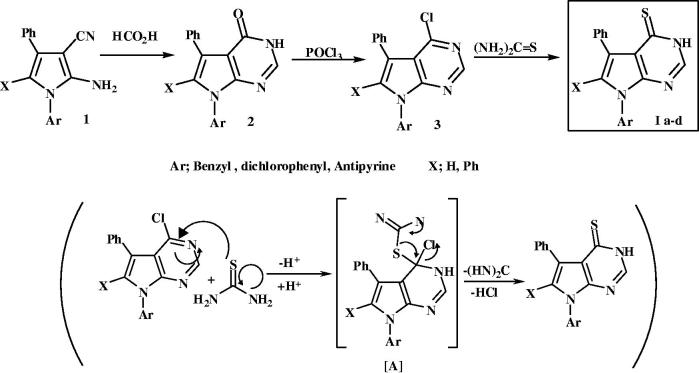
Synthetic and mechanistic pathway for preparation of Pyrrolopyrimidine-4-thione **Ia–d**.

The synthetic route to compounds **Ia**–**d** was reported in our previous work^40–42^. Amino-cyano-pyrroles **1** were reacted with HCO_2_H to produce pyrrolopyrimidin-4-ones **2**, which on react with POCl_3_, 4-chloro-pyrrolopyrimidines **3** were obtained in good yield. 4-Chloro derivatives **3** on react with thiourea adapted to pyrrolopyrimidin-4-thiones **I**, To date, and to the best of our knowledge, formation of the 4-thione analogues has been reported numerously in literature^29^^,^^32^^,^^43^^,^^44^; but not mechanistically explained. Herein, the proposed mechanism of the reaction was believed to proceed *via* initial nucleophilic attack by the thiol group of thiourea on C-4 of the pyrimidine ring with proton transfer to N-3 and the formation of the potentially unstable intermediate [A]. This intermediate lose carbodiimide and HCl to give the pyrrolopyrimidin-4-thione, as revealed (Figure 4).

For preparation of spiro-pyrrolopyrimidines **III**–**V**; pyrrolopyrimidin-4-ylidene-malononitrile **IIa**–**d** was accomplished by the reaction of **Ia**–**d** with malononitrile in absolute ethanol using same procedure reported on our previous work^45^. On treat thione derivatives **II** with acetylacetone guanidine hydrochloride and/or hydrazine hydrate in ethanol, containing catalytic amount of pyridine, the corresponding spiro-pyrrolopyrimidine derivatives of pyrazole, pyrimidine or pyran **III**–**V** were afforded in good yield, as revealed in Scheme 1. All novel compounds were confirmed with spectroscopic analysis (MS, IR, ^1^H NMR and microanalysis).

### Biological activities

Twelve of synthesised spiro-pyrrolopyrimidines and pyrrolopyrimidine-4-one were evaluated for their anti-hyperglycemic activity using both sucrose load model and streptozotocin models of diabetes^7^^,^^36^^,^^46–49^. The synthesised compounds were assessed for their anti-hyperglycemic activity, which is comparable to Glimepiride (Amaryl) the standard anti-hyperglycemic drug, by comparing the mean area under the curve (AUC) for the blood glucose level between the different studied groups.

**Figure 5.**
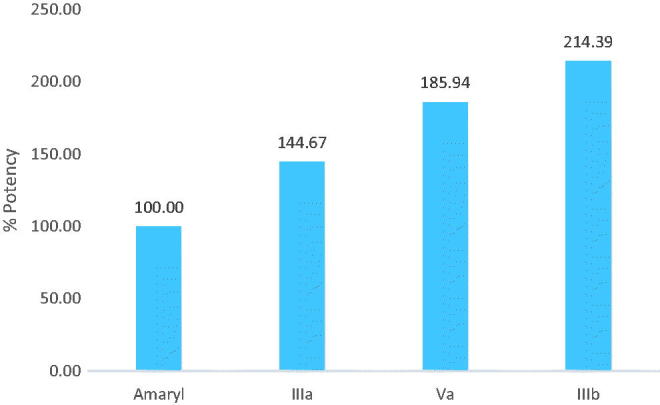
Potency of anti-hyperglycemic derivatives compared to Amaryl.

Among the 12 tested compounds; five compounds showed significant improvement (12.32%, 13.3%, 14.52%, 15.18% and 21.54%, respectively) on oral glucose tolerance post-sucrose-loaded normoglycemic rats compared to the sucrose-loaded untreated control, as revealed in Figure 5. From those active derivatives, treatment of derivatives **IIIa**, **IIIb** and **Va** only to STZ model of diabetes caused lowering on the blood glucose profile to the average of (17.49%, 22.48% and 25.92%, respectively) compared to the diabetic control group, as depicted in Table 1.

**Table 1.** Assessment of various treatments on oral glucose tolerance and blood glucose lowering activity in sucrose load model and diabetic rats.

Tested compounds	Mean (AUC ± SEM)		Reduction in blood glucose compared to control (%)	
	SLM	STZ	SLM	STZ
Amaryl	88.2 ± 5.3***	1373.64 ± 49.55*	60.97***	12.09*
**IIa**	191.6 ± 3**	**NA**	15.18**	**NA**
**IIb**	177 ± 12.2**	**NA**	21.54**	**NA**
**IId**	214.4 ± 5.2	—	NA	—
**IIIa**	198.2 ± 3.1**	1289.16 ± 90.16*	12.32**	17.49*
**IIIb**	193.2 ± 2.6*	1157.5 ± 102.31*	14.52*	25.92*
**IIIc**	222.3 ± 8.6	—	NA	—
**IIId**	203.8 ± 6.5	—	NA	—
**IVa**	223.3 ± 7.7	—	NA	—
**Va**	195.9 ± 2.2**	1211.20 ± 89.86*	13.3**	22.48*
**Vb**	207.4 ± 4.6	—	NA	—
**Vc**	220 ± 12.8	—	NA	—
**VIa**	204 ± 9.2	—	NA	—

* *p* < .05, **p < .01 and ***p < .001 significant different compared to control group (active compounds).

Values were expressed as mean ± SEM. Using parametric unpaired *t*-test. NA: not active; SLM: sucrose-loaded model; STZ: streptozotocin model of diabetes.

**Figure 6.**
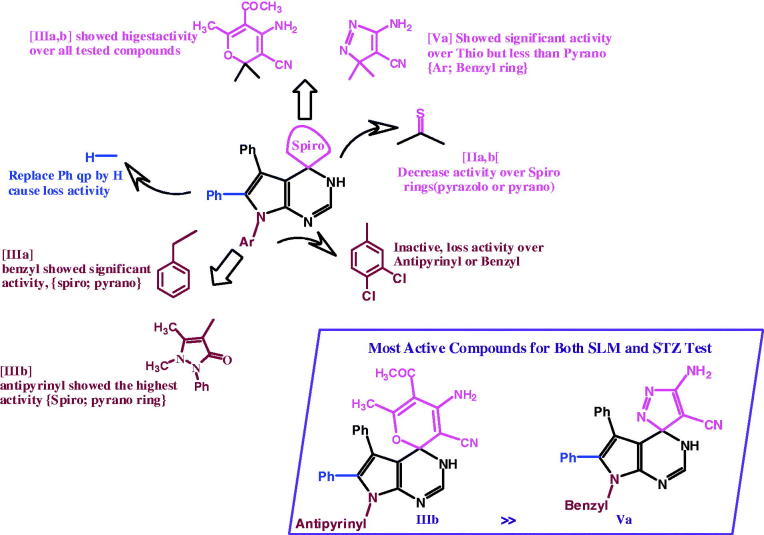
Active compounds structural analysis and discussion.

Comparing the anti-hyperglycemic activity of these compounds to that of the reference anti-diabetic drug (Amaryl), compounds **IIIa**, **Va** and **IIIb** showed significant decrease in the blood glucose level (144.67%, 185.94% and 214.39%, respectively) compared to the activity of Amaryl, as shown in Figure 6. Studying these anti-hyperglycemic derivatives **IIIa**, **Va** and **IIIb** showed that the rats survived and showed no toxicity symptoms, as revealed in Table 1.

Active compounds were classified into two main sets: first, the 4-malononitrile derivative of pyrrolopyrimidines, namely, **IIa,b** (Ar = benzyl and anti-pyrine). Also, the spiro derivatives containing pyrane ring **IIIa,b**, spiro-containing pyrazole ring **Va**.

## Conclusions

We designated a direct and efficient synthesis of novel spiro-pyrrolopyrimidine, and estimated as anti-hyperglycemic agents. The structure activity analysis indicated that the pyrano **IIIa,b** displayed a significant anti-hyperglycemic activity profile compared to Amaryl. Pyrimidine group in **IVa** did not enrich the activity. The introduction of pyrazolo group to **Va** give rise to superior anti-hyperglycemic activity.

## Supplementary Material

IENZ_1461854_Supplementary_Material.pdf
