# Supplementary figures and images for: Synthesis and evaluation of novel spiro derivatives for pyrrolopyrimidines as anti-hyperglycemia promising compounds
Source: J Enzyme Inhib Med Chem. 2018 Apr 30;33(1):809–17. doi: 10.1080/14756366.2018.1461854 (PMC6009929; doi:10.1080/14756366.2018.1461854)

## Supplementary Material

### Spiropyrimidines as anti-hyperglycemic agents

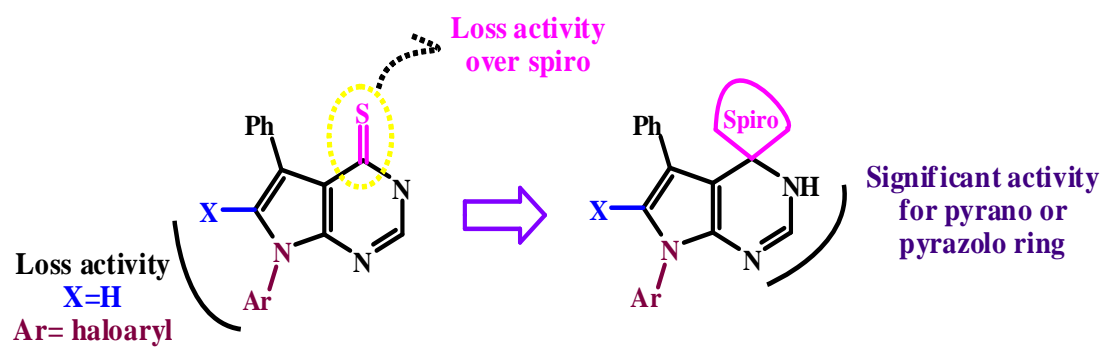

Supplement: IENZ_1461854_Supplementary_Material.pdf [file IENZ_A_1461854_SM5518.pdf]
